# Round cell sarcoma of the colon with CIC rearrangement

**DOI:** 10.1186/s13104-017-2906-0

**Published:** 2017-11-09

**Authors:** H. Maghrebi, R. Batti, A. Zehani, N. Chrait, H. Rais, A. Makni, A. Haddad, M. Ayadi, A. Daghfous, M. Jrad, N. Kchir, Z. Bensafta, A. Mezlini

**Affiliations:** 1 Surgery Department la Rabta Hospital Tunis, Tunis, Tunisia; 2 Oncology Unit, Institut Salah Azaiez Tunis, Tunis, Tunisia; 3 Pathology Department la Rabta Hospital. Tunis, Tunis, Tunisia; 4 Radiology Department la Rabta Hospital. Tunis, Tunis, Tunisia; 5 Faculty of Medicine of Tunis, University of Tunis El Manar, Tunis, Tunisia

**Keywords:** Sarcoma, Round cell, Translocation, Genetic, Surgery

## Abstract

**Background:**

The *CIC*-*rearranged* sarcoma is a very rare highly aggressive malignant soft tissue group of tumors. It has recently been described as highly aggressive soft tissue tumors of children and young adults sharing similar morphological features with the Ewing sarcoma. The digestive localization is exceptional.

**Case presentation:**

A 14-year-old male presented with a history of abdominal pain for 1 year, which increased in intensity over the last 2 months. Imaging findings showed a large heterogeneous mesenteric mass on the left flank of the abdomen. Exploratory laparotomy was performed and revealed a large cystic hypervascularized mass depending on the transverse colon and mesocolon. A wide excision of the lesion was performed with segmental colectomy. No postoperative complications were noted. The microscopic examination revealed a vaguely nodular growth of undifferentiated small round cells, arranged in solid sheets separated by thin fibrous septa with a scarce stroma. After an uncomplicated post-operative course, the patient was referred for chemotherapy. The patient died 2 months later with a peritoneal and pleural progression.

**Conclusions:**

The *CIC*-*rearranged* sarcoma is an aggressive tumor. There is no standard therapy for this rare disease. Their treatment includes surgery and chemotherapy. Resistance to chemotherapy is common. Further publications and studies will help to determine a standard therapy for this rare disease.

## Background

Round cell sarcoma is a very rare highly aggressive malignant soft tissue group of tumors. They represent 1.1% of all malignant soft tissue tumors [1]. The digestive localization is exceptional [2]. *CIC* rearrangements have been reported in soft tissue tumors [3]. They share morphological similarities with the more common Ewing sarcoma.

We report herein the first case of *CIC*-*rearranged* sarcoma arising from the colon and describe the presenting symptoms, imaging findings, pathological features and molecular genetics of the tumors.

## Case presentation

A 14-year-old male presented with a history of abdominal pain for 1 year, which increased in intensity over the last 2 months. On examination there was sensitivity on the left abdominal quadrant. An ultrasound scan revealed a complex mass evoking an hematoma.

**Fig. 1:**
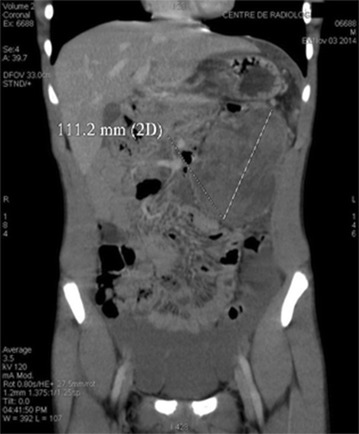
Computed tomography scan showing the mass

**Fig. 2:**
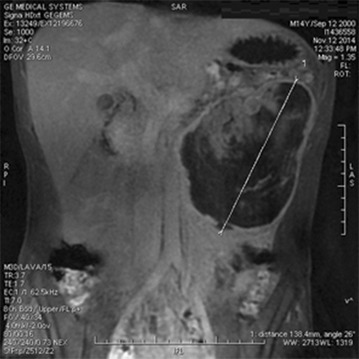
Magnetic resonance imaging (MRI) of the abdominal mass

**Fig. 3:**
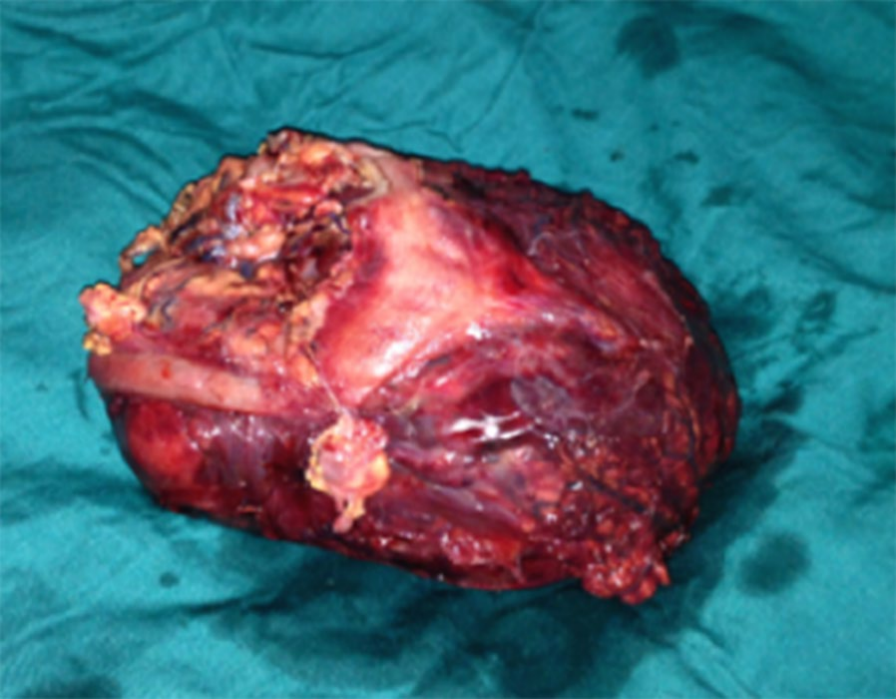
Peroperative view of the mass

The computed tomography scan (CT scan) (Fig. 1) and magnetic resonance imaging (MRI) (Fig. 2) showed a large heterogeneous mesenteric mass on the left flank, measuring 110 × 86 mm, with internal vegetation and superior polar tissular component. It has a very close contact with the colon stomach and lower edge of the pancreas. Neither adenopathy nor other lesions were found. However, there was no evidence of distal metastatic spread. Tumor markers, such as carcino-embryonic antigen (CEA) and carbohydrate antigen *19*-*9 (CA 19*-*9)*, were normal. Exploratory laparotomy was performed and revealed a large cystic hypervascularized mass depending on the transverse colon and mesocolon (Fig. 3).

A wide excision of the lesion was performed with segmental colectomy. No postoperative complications were noted. The patient was discharged from the hospital in good health on the 7th postoperative day.

Pathology examination showed on macroscopy a heterogeneous semi-solid cystic mass of the mesocolon without any involvement of the colic wall. The mass contained extensive hemorrhagic necrosis.

**Fig. 4:**
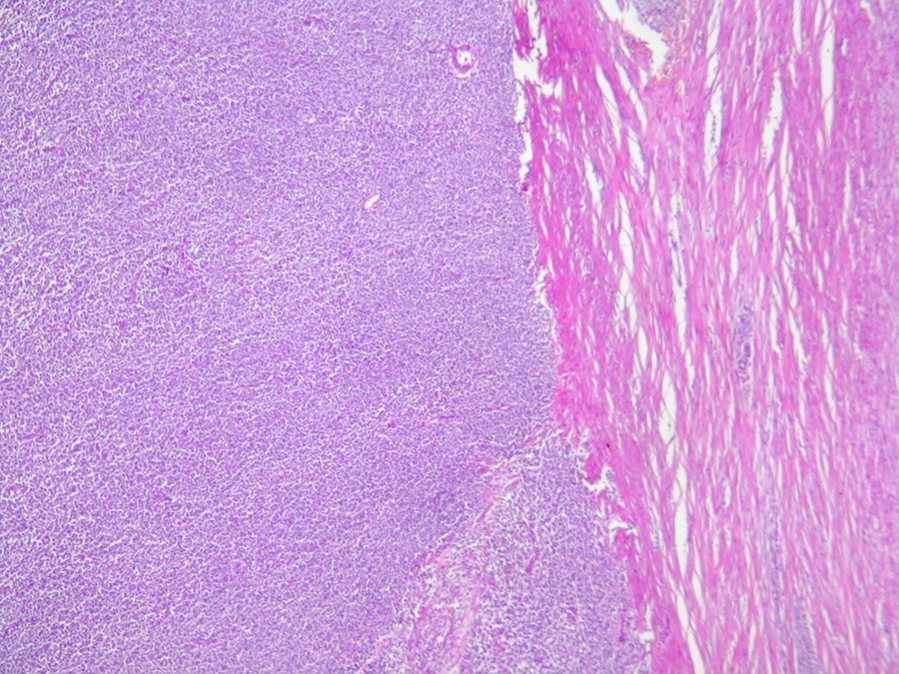
(Hematoxylin–eosin): undifferentiated tumor infiltrating colonic wall

**Fig. 5:**
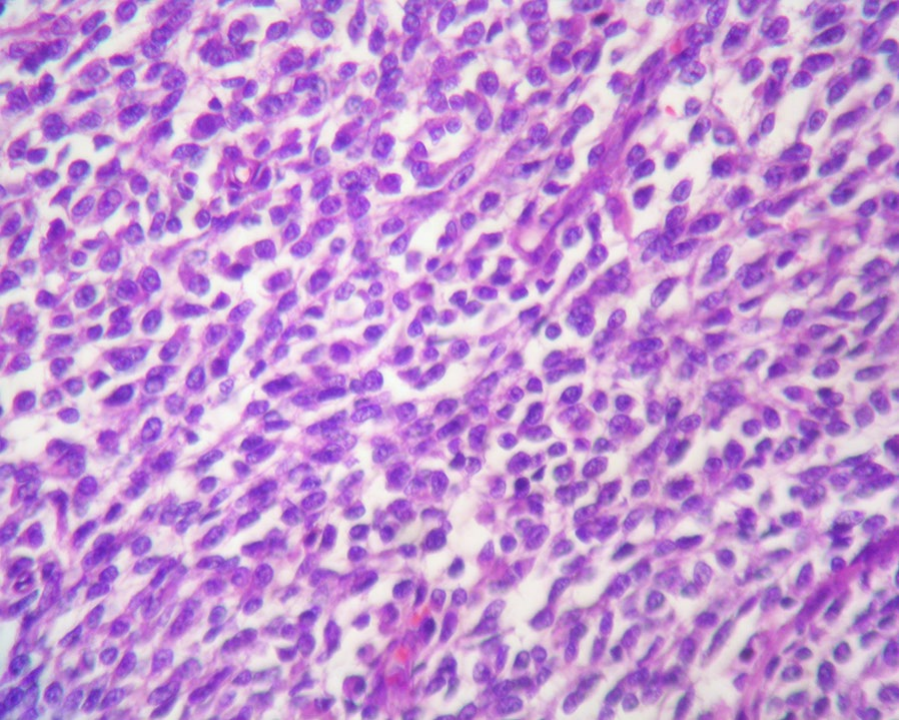
(Hematoxylin–eosin): round tumor cells with eosinophilic cytoplasm and mild pleomorphism

**Fig. 6:**
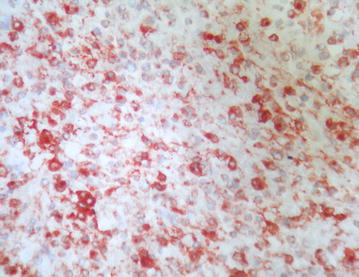
Immunohistochemistry (tumor cells were positive for CD99)

The microscopic examination (Figs. 4, 5, 6) revealed an undifferentiated tumor infiltrating the colonic wall The tumor was composed of sheets of round small cell tumors divided by fibrous septa. Tumor cells had an ill defined cell borders with scant eosinophilic cytoplasm. The nuclei showed mild pleomorphism, vesicular chromatin and prominent nucleoli. A high mitotic rate of 25 per 10 high-power fields was detected. Geographic areas of necrosis were seen. In immunohistochemistry the tumor cells showed diffuse positivity for ETV4 and CD99. Keratin AE1/AE3, S100 protein, EMA, CD34, desmin and myogenin were negative. The tumor cells were also negative for lymphoid markers, Dog1 and cyclin B3. Fluorescence in Situ Hybridization (FISH) analysis revealed a rearrangement of the *CIC* gene.

The mesenteric lymph nodes were free. The surgical margins of the resection were clean. The final diagnosis was a *CIC*-*rearranged* sarcoma. A whole-body bone scan revealed no abnormal increased uptakes and a thoracic CT scan did not detect additional metastases. After an uncomplicated post-operative course, the patient was referred for chemotherapy by vincristine, ifosfamide, doxorubicin and etoposide associated to growth-clony stimulating factors (G-CSF). Nevertheless, the patient developed a neutropenic fever at the first cycle; hence the dose reduction in the next courses. The CT scan at the 4th course revealed a peritoneal progression with nodules in paracolic gutters and the right upper abdomen. The greatest nodule was in the Douglas cul-de-sac and measured 8.5 cm. The patient underwent two courses of temozolamide–irinotecan. He developed peritoneal progression with pleural metastasis (without lung metastasis). He died 2 months later.

## Discussion

The *CIC*-*rearranged* sarcoma is a very rare highly aggressive malignant soft tissue group of tumors. It is currently being recognized as emerging entities. It is a huge family of undifferentiated round cells [4]. Recent molecular advances, especially next generation sequencing, have allowed identification of a number of subtypes of undifferentiated round cell. *CIC* rearrangement sarcomas have recently been described as highly aggressive soft tissue tumors of children and young adults sharing similar morphological features with the Ewing sarcoma. It shows an aggressive course with early metastasis and poor prognosis [5, 6].

Round cell tumors can be considered as a differential diagnosis especially Ewing sarcoma family, alveolar rhabdomyosarcoma or poorly differentiated synovial sarcoma. Diagnosis requires radiology, histopathology, IHC and cytogenetic studies [7–10].

The gene capicua transcriptional repressor (*CIC*), located on the chromosome *19q13.2*, is a gene involved in the way of the EGFR and is over-expressed in the cerebellar tissue.

In a recent review of Specht et al. [11], the digestive localization is still exceptional. The authors reported only two cases arising from the stomach and from peritoneum.

Despite aggressive treatment, they exhibit high rates of metastasis to lung and brain, often with a poor prognosis, and median survival is < 2 years.

There is no standard therapy for this rare disease. It’s often treated “like Ewing sarcoma”, by surgery and chemotherapy. Chemotherapy combinations such as doxorubicin and ifosfamide, or doxorubicin, vincristine and cyclophosphamide are usually used.

## Conclusion

Undifferentiated small round cell sarcomas are aggressive tumors. Their treatment includes surgery and chemotherapy. Resistance to chemotherapy is common. Lung and brain are common sites of metastasis, with poor prognosis. Generally, median survival is less than 2 years. Newer techniques have been developed recently which helped identify a subset of previously unclassifiable sarcomas, with promising prognostic value.

## Abbreviations

CT scan: computed tomography scan
MRI: magnetic resonance imaging
CEA: carcino-embryonic antigen
CA 19-9: carbohydrate antigen 19-9
IHC: immunohistochemistry
G-CSF: growth-clony stimulating factors
PNET: primitive neuroectodermal Tumor
CIC: capicua transcriptional repressor
